# Gender Differences in Moderate Drinking Effects

**Published:** 1999

**Authors:** Martin S. Mumenthaler, Joy L. Taylor, Ruth O’Hara, Jerome A. Yesavage

**Affiliations:** Martin S. Mumenthaler, Ph.D., is a research associate; Joy L. Taylor, Ph.D., is a research associate; Ruth O’Hara, Ph.D., is a senior research scholar; and Jerome A. Yesavage, M.D., is a professor at the Department of Psychiatry and Behavioral Sciences, Stanford University School of Medicine, Stanford, California

**Keywords:** moderate AOD use, gender differences, ethanol metabolism, AOD impairment, pharmacokinetics, female, nervous system, BAC, liver, dose response relationship, memory, attention, CNS information processing, psychomotor impairment, sex hormones, menstrual cycle, literature review

## Abstract

Women appear to become more impaired than men after drinking equivalent amounts of alcohol, achieving higher blood alcohol concentrations even when doses are adjusted for body weight. This finding may be attributable in part to gender differences in total body water content. Men and women appear to eliminate approximately the same total amount of alcohol per unit body weight per hour. However, women seem to eliminate significantly more alcohol per unit of lean body mass per hour than men. Some studies report that women are more susceptible than men to alcohol-related impairment of cognitive performance, especially in tasks involving delayed memory or divided attention functions. Psychomotor performance impairment, however, does not appear to be affected by gender. This article provides an overview of alcohol metabolism (pharmacokinetics) and reviews recent studies on gender differences in alcohol absorption, distribution, elimination, and impairment. Speculation that gender differences in alcohol pharmacokinetics or alcohol-induced performance impairment may be caused by the menstrual cycle and variations in female sex hormones are discussed. It is concluded that the menstrual cycle is unlikely to influence alcohol pharmacokinetics.

Research has confirmed the observation that women become more impaired than men after drinking similar quantities of alcohol. In addition, women appear to be more susceptible than men to alcohol’s long-term health effects (e.g., alcoholic liver disease). The prevalence of chronic alcohol-related problems is significantly lower among women, however, perhaps in part because only 2 percent of American women are heavy drinkers,^1^ compared with 9 percent of men (Substance Abuse and Mental Health Services Administration 1998). Studies report adverse effects in men and women at even moderate drinking levels.^2^ Among those effects are disturbances of sensory information processing, short-term memory, reaction time, and eye-hand coordination. These deficits can impair the ability to drive a motor vehicle and may persist to the following day, impeding one’s performance at work. The potential influence of gender on the acute effects of alcohol is therefore of importance for the large number of women who are social drinkers.

This article reviews research on the differential effects of moderate drinking on men and women and the mechanisms that may underlie these differences. These mechanisms fall into two categories: (1) gender differences in the physiological processing and elimination of alcohol (i.e., alcohol pharmacokinetics) and (2) differential sensitivity of the nervous system to alcohol’s effects. Finally, the article evaluates data on the influence of the menstrual cycle and female reproductive hormones on the pharmacokinetics and nervous system effects of alcohol.

## Overview of Alcohol Pharmacokinetics

Blood alcohol concentration (BAC) is determined by the rate of alcohol absorption from the gastrointestinal (GI) tract into the bloodstream, the volume of distribution in the body, and the rate of elimination. Absorption and distribution determine the proportion of an ingested drug or other chemical substance that reaches the organs (alcohol bioavailability), where it may subsequently exert its effects. Alcohol is eliminated from the body largely by a metabolic process called oxidation, which occurs mostly in the liver. Some oxidation of alcohol also occurs during the absorption phase, thereby affecting the bioavailability of alcohol.

### Absorption and Distribution

Alcohol consumed by mouth is rapidly absorbed into the bloodstream from the stomach and small intestine. The rate of absorption of alcohol depends on several factors, including the amount and concentration of alcohol ingested and the quantity and composition of food in the stomach. Alcohol absorbed from the small intestine flows through the portal vein directly to the liver, where a portion of the alcohol is metabolized. The process by which a substance is metabolized before entering the general circulation is called first-pass metabolism (FPM).

Many toxic substances undergo hepatic FPM. In the first step of hepatic FPM of alcohol, an oxidative enzyme called alcohol dehydrogenase (ADH) converts alcohol into acetaldehyde.^3^ Hepatic FPM is most significant with low to moderate alcohol doses of about 0.4 grams per kilogram of body weight (g/kg), which is equivalent to about two standard drinks^4^ for a person weighing 70 kg (approximately 150 pounds). The extent of FPM in the liver depends on the rate of absorption of alcohol from the GI tract. If absorption is fast, the quantity of alcohol reaching the liver can exceed the metabolic capacity of available ADH. This allows a greater proportion of alcohol to escape FPM and reach the general circulation, resulting in a higher peak BAC. Conversely, slow absorption leads to a higher FPM and a lower peak BAC. For example, the presence of food in the stomach leads to slower alcohol absorption, hence more efficient FPM and a lower peak BAC. The time from last drink to peak BAC usually ranges from 30 to 90 minutes (Hardman and Limbird 1995).

Some FPM may occur in the GI tract (mostly in the stomach) as well as in the liver (Yin et al. 1997). Although earlier research reported decreased gastric ADH activity in women compared to men (Frezza et al. 1990), the relative contribution of gastric ADH activity to overall FPM in both genders is controversial. One study found the activity of gastric ADH in the rat to be approximately 14 percent that of hepatic ADH (Lee et al. 1992). Another study estimated that gastric FPM in humans accounts for 0.4 percent of a low alcohol dose, compared with 4 percent metabolized in the liver (Derr 1993).

The portion of alcohol that is absorbed from the GI system and that escapes FPM enters the general circulation and is rapidly distributed throughout the body, preferentially in body water (i.e., within the bloodstream and in the fluid within and between cells). Studies on alcohol pharmacokinetics must therefore take into account subjects’ body compositions, a significant factor in gender-related studies.

### Elimination

Alcohol can be oxidized by ADH in various organs, including the stomach and small intestine. Quantitatively, however, most alcohol metabolism occurs in the liver. Between 90 and 98 percent of alcohol that enters the body is eventually completely oxidized. The rate of alcohol metabolism is related to blood alcohol concentration (BAC). At BACs below approximately 0.02 percent,^5^ the rate of alcohol metabolism is exponential. At higher BACs, the functional capacity of the ADH system becomes saturated, and the alcohol elimination rate remains relatively constant between approximately 0.020 and 0.065 percent BAC. The rate of elimination of larger doses of alcohol increases as BAC increases (Holford 1987; Lundquist and Wolthers 1958).

A secondary pathway of alcohol metabolism exists called the microsmal ethanol oxidizing system (MEOS). At low to moderate BACs, the role of MEOS appears to be minor compared with that of ADH. Large doses of alcohol accelerate MEOS activity, however, possibly accounting for the increased rate of alcohol metabolism that accompanies increased BACs (Lieber 1997).^6^

## Gender Differences in Alcohol Pharmacokinetics

Significant gender differences in alcohol pharmacokinetics appear to include increased bioavailability and faster disappearance rates in women. Some relevant studies are summarized below.

### Increased Alcohol Bioavailability in Women

Women have proportionally more body fat and less water than do men of the same body weights. Because alcohol is dispersed in body water, women reach higher peak BACs than men after consuming equivalent doses of alcohol, even when doses are adjusted for body weight (Frezza et al. 1990; Taylor et al. 1996). In one study, gender differences in BAC disappeared when equivalent doses were administered based on total body *water* (Goist and Sutker 1985).

Higher peak BACs in women may also reflect lower rates of FPM by gastric (Frezza et al. 1990; Vogel-Sprott 1992) or hepatic (Thomasson 1995) ADH. In one study, however, six men and six women consumed a moderate dose of alcohol (0.3 g/kg ) after a standardized meal; in this study, each person’s total FPM was estimated at 5 to 14 percent of the ingested dose, with no significant difference between genders (Ammon et al. 1996). Another study found that gastric ADH activities did not differ significantly between men and women (Yin et al. 1997). Thus, although the effect of gender on FPM remains controversial, the most recent data have failed to confirm a gender effect on FPM via ADH activity.

### Faster Alcohol Disappearance Rates in Women

A pharmacokinetic elimination measure that is not influenced by differences in total body water (TBW) is alcohol disappearance rate (β_60_), which is defined as the rate of decrease of BAC during the linear (i.e., concentration-independent) phase of elimination, expressed in grams of alcohol per liter of blood per hour (g/L/h). Alcohol disappearance rate should be the preferred elimination measure for pharmacokinetic cross-gender comparisons, where different body composition is one of the major aspects that prevents equal dosing.

Of 13 studies measuring gender differences in terms of β_60_ using moderate alcohol doses (0.3 to 0.8 g/kg), 9 concluded that women reach higher β_60_ than do men, whereas 4 studies found no significant gender differences in β_60_ (see table 1). Eight of the nine most recent studies found significantly higher β_60_ in women compared with men. No gender differences were found in two studies that measured alcohol elimination rates (AERs) in terms of g/kg/h (Marshall et al. 1983; Sutker et al. 1983).

Liver volume has been found to correlate directly with alcohol metabolic rate in rats (Lumeng et al. 1979). Equivalent AERs in terms of eliminated grams of alcohol per hour between genders might therefore be explained by the finding of a higher liver volume per unit lean body mass in women compared with men (Li et al. 1998; Kwo et al. 1998). Kwo and colleagues (1998) detected no significant difference in mean AER and mean computed liver volume between young men and women. However, lean body mass was 42-percent greater in men than in women. Thus, compared with men, women had a 33-percent higher mean AER and a 38-percent higher liver volume per kg lean body mass. These results might explain the finding (Taylor et al. 1996) that compared with men, women reached significantly higher β_60_ (in g/L/h, representing AER per kg lean body mass in the study of Kwo and colleagues), whereas AERs (in g/kg/h) were not significantly different between genders (Kwo et al. 1998).

In sum, evidence suggests that men and women eliminate approximately the same total amount of alcohol per hour but that women reach significantly greater clearance of alcohol per unit of lean body mass compared with men (Eckardt et al. 1998) and thus reach significantly greater alcohol disappearance rates (in g/L/h). In other words, women eliminate more alcohol *per volume of blood* per hour than do men.

Several possible explanations exist for existence of higher β_60_ rates in women. For example, higher peak BACs in women might accelerate alcohol metabolism by activating MEOS. However, a study that achieved comparable peak BACs for both men and women (i.e., 0.08 percent) by applying a 12.5-percent dose reduction for women also showed faster β_60_ in women (see figure) (Taylor et al. 1996). Some evidence suggests that slower alcohol disappearance in men may reflect inhibition of alcohol metabolism by the male reproductive hormone dihydrotestosterone. For example, dihydrotestosterone appears to inhibit hepatic ADH in rats (Mezey and Potter 1982) and decrease liver alcohol dehydrogenase content in humans (Mezey et al. 1988). The possible role of female reproductive hormones is discussed in a later section.

## Gender Differences in Dose-Related Impairment

Moderate alcohol consumption can impair cognition (e.g., divided attention, information processing, reaction time, and memory) and psychomotor performance (e.g., eye-brain-hand coordination and body sway). More complex cognitive and psychomotor performance tasks are more sensitive to alcohol’s effects than are simpler tasks (Eckardt et al. 1998), and alcohol-induced impairment can occur at levels well below 0.05 percent (Hindmarch et al. 1991). The ability to divide attention between two or more sources of visual information is particularly important when driving a motor vehicle or flying an airplane. Divided attention deficits have been shown at BACs as low as 0.015 percent (Moskowitz and Burns 1990).

Table 2 summarizes results of some studies on gender differences in post-drink performance. Niaura and colleagues (1987) evaluated 11 men and 13 women as they performed cognitive and psychomotor tasks, including divided attention, short-term memory, and pursuit tracking,^7^ both while sober and at BACs adjusted to 0.054 percent for men and 0.062 percent for women. No overall gender differences were detected in intoxicated performance when gender differences in BACs were controlled statistically. However, women recovered short-term memory functioning significantly more slowly than did men. This finding confirmed an earlier study at BACs of 0.04 percent in which alcohol affected women significantly more than it affected men on a similar short-term memory task (Jones and Jones 1977). Another investigation showed that women were significantly more impaired than men on delayed recall when intoxicated with moderate doses of alcohol (i.e., BACs of 0.063 percent in men and 0.072 percent in women). This study, however, failed to detect gender differences on immediate recall (Jones and Jones 1976*a*, *b*).

Retrieval from long-term memory also appears to be affected differently between genders. When intoxicated, women respond significantly more slowly on cognitive decision tasks than do men (Haut et al. 1989).

**Figure:**
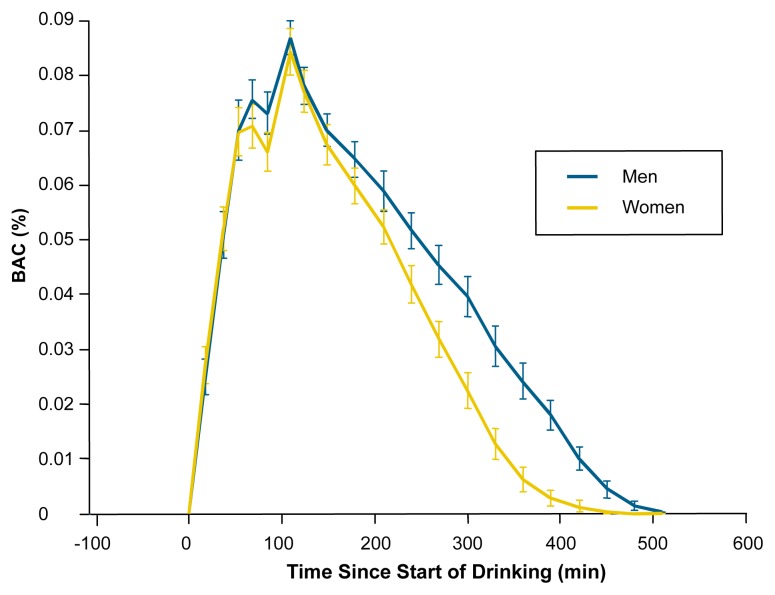
Gender differences in blood alcohol concentration (BAC) over time. A total of 11 men and 12 women were administered alcohol over a period of 90 minutes (min). Although women received 12.5 percent less alcohol per kilogram of body weight than did men, both genders achieved an average peak BAC of approximately 0.08 percent. Measurements taken over the next 8 hours showed a more rapid decline of BAC in women than in men, indicating a faster alcohol disappearance rate in women. (From Taylor et al. 1996, with permission.)

Gender differences are more apparent at higher alcohol doses. On a task that measured divided attention performance, women showed significantly greater impairment than men after consuming alcohol doses sufficient to produce equivalent moderate BACs of 0.06 percent. Conversely, no gender differences in cognitive impairent were measurable after lower doses producing equivalent BACs of 0.03 percent (Mills and Bisgrove 1983).

Short-term memory, information processing, and divided attention are especially critical in aviation. Flying an airplane demands a high level of alertness, three-dimensional thinking, and psychomotor coordination as well as the simultaneous performance of many subtasks that compete for limited processing capacity. Modern flight simulators provide a precise instrument of measuring cognitive ability and sensory-motor functioning and therefore serve as an ideal tool for studying the effects of low to moderate doses of alcohol.

Taylor and colleagues (1996) investigated gender-related differences in response to alcohol doses in a flight simulator. Alcohol doses were adjusted to yield average peak BACs of 0.087 percent for women and 0.084 percent for men. There was a significant impairment for both genders in flight performance during acute intoxication but not 8 hours after alcohol administration, when BACs had returned to zero. No significant gender differences in overall impairment were detected, despite the fact that women showed faster disappearance rates and reached a BAC of 0.04 percent^8^ approximately 1 hour before men.

This finding suggests that in highly skilled subjects, gender does not influence alcohol’s effects on sustained performance of multiple tasks. Conversely, as previously mentioned, alcohol impairment on some single short-term *cognitive* tasks does appear to be influenced by gender. Several other studies that tested *psychomotor* performance tasks, such as dart throwing, hand steadiness, and body sway, found no influence of gender on alcohol-induced performance impairment (Burns and Moskowitz 1978; Mills and Bisgrove 1983; Niaura et al. 1987; Wait et al. 1982).

## Influence of the Menstrual Cycle

### Menstrual Cycle Effects on Alcohol Pharmacokinetics

As a possible explanation for gender differences in alcohol pharmacokinetics, researchers have suggested that the physiological responsiveness of women to alcohol may vary throughout the menstrual cycle as a result of changes in levels of sex steroid hormones (see sidebar on p. 62). The mechanism for such a possible effect is unclear. Researchers have speculated that estrogens and progesterone may influence hepatic ADH activity or other stages of alcohol metabolism (Lammers 1995).

**The Menstrual Cycle**

Women of childbearing age exhibit rhythmic changes in the production of reproductive hormones. This pattern, commonly referred to as the menstrual cycle, generally averages 28 days. The timing of the cycle is regulated by complex interactions among many hormones and other chemical messengers produced in the brain, reproductive organs, and other tissues. The hormones of primary interest in this article belong to a class of substances called steroids, which are synthesized within the body from dietary cholesterol.

The principal female sex steroids, progestrone and estrogens,^1^ are produced largely in the ovaries and uterus. Among other functions, estrogens stimulate the proliferation and growth of cells within the sexual organs. Progesterone primarily facilitates pregnancy and lactation.

The menstrual cycle is considered to begin with menstruation. The midpoint of the cycle is characterized by ovulation, in which a single egg cell (ovum) is expelled from the ovary. If the ovum is fertilized by a sperm, it lodges in the uterus and develops into a fetus.

**Figure:**
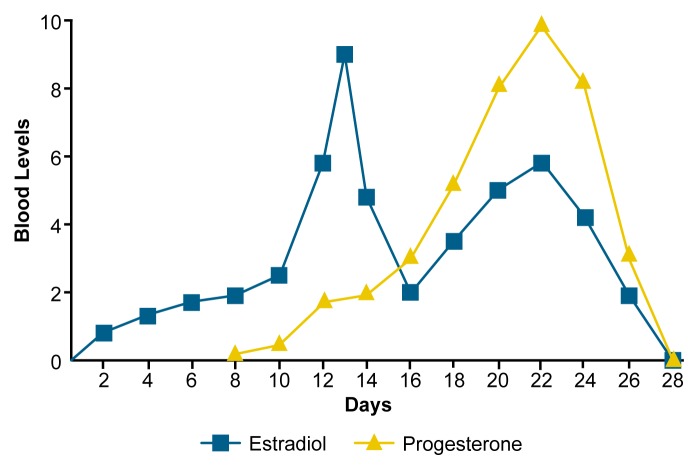
Blood levels of the major female steroid reproductive hormones throughout the menstrual cycle. Concentrations are expressed in the following units: for progesterone, nanograms of hormone per milliliter of blood (ng/mL); for estradiol, thousands of ng/mL. Estradiol is the primary estrogen in humans. Day 1 represents the onset of menstruation. SOURCE: Adapted from Broverman, D.M.; Vogel, W.; Klaiber, E.L.; Majcher, D.; Shea, D.; and Paul, V. Changes in cognitive task performance across the menstrual cycle. *Journal of Comparative and Physiological Psychology* (now known as the *Journal of Comparative Psychology*) 95:646–654, 1981.

***Phases of the Menstrual Cycle***

The menstrual cycle has five phases, as described below.

*Menstrual Phase (Days 1–5).* Menstrual flow occurs if the expelled ovum has not become fertilized during the ovulatory phase (see below). Levels of progesterone and estrogens are low.

*Follicular Phase (Days 6–12)*. The ovary secretes small but increasing quantities of estrogens, stimulating the development within the uterus of specialized cell clusters called follicles. Each follicle nurtures a developing ovum and further contributes to the secretion of estrogens. Only one follicle reaches full maturity.

*Ovulatory Phase (Days 13–15)*. The mature follicle ruptures, releasing its ovum. Fertilization can occur at this time.

*Luteal Phase (Days 16–23)*. The empty follicle becomes a glandlike structure called the corpus luteum, which secretes large quantities of estrogens and progesterone. These hormones prepare the uterine lining for implantation of the fertilized ovum.

*Premenstrual Phase (Days 24–28)*. If the ovum is not fertilized, the corpus luteum degenerates and stops secreting progesterone and estrogens. Decreased levels of these hormones result in the shedding of the uterine lining (i.e., menstruation). If pregnancy occurs, the corpus luteum remains intact and secretes increasing quantities of steroid hormones, promoting the development of the fetus and the progression of pregnancy.

***Summary***

Levels of both estrogen and progesterone are low from day 28 to day 7 (i.e., during menstruation) and high between days 19 and 25. During days 12 to 14, estrogen levels are high, whereas progesterone levels are low (see figure).

— John J. Doria

1The primary estrogen in humans is known as estradiol.

Of 18 studies reviewed, 5 found menstrual cycle effects on alcohol pharmacokinetics (see table 3). The results of these studies are conflicting. In one study, women tested during the premenstrual phase reached higher peak BACs and faster alcohol absorption rates than did women at other phases of the menstrual cycle (Jones and Jones 1976*a*). However, the same researchers who conducted that study failed to replicate those results in a later study (Jones and Jones 1984). In other studies, women tested during the premenstrual phase (i.e., day 26 of the menstrual cycle) reached lower peak BACs than during menstruation (i.e., day 1), and women tested on day 24 reached lower peak BACs and alcohol disappearance rates than during day 1 (Kegg and Zeiner 1980; Zeiner and Kegg 1981). Sutker and colleagues (1987*b*) found that the alcohol disappearance rate during the luteal phase (i.e., days 20 to 25) was 14 percent higher than during the early follicular phase (i.e., days 2 to 7) and 8 percent higher than during the ovulatory phase (i.e., day 14). However, significant differences in elimination across the menstrual cycle did not occur until BACs dropped below 0.02 percent; thus, the practical importance of that finding seems to be suspect.

Two recent reviews (Gill 1997; Lammers 1995) identified several methodological limitations of the prior investigations. First, the number of subjects in these research studies is usually small. Therefore, to achieve statistical significance, studies should employ a within-subjects design (WSD), in which each subject serves as her own control and is tested at various time points during the menstrual cycle. Second, if variations in pharmacokinetics are to be meaningfully related to estrogen or progesterone activity, the time points selected for testing should be characterized by significant variations in sex steroid levels, as confirmed by blood measurements. Finally, the occurrence of ovulation should be verified by measuring sex steroid levels. For example, Metcalf and Mackenzie (1980) found that 25 percent of normally cycling women between the ages 20 and 39 had at least one cycle without ovulation during a 3-month period. When ovulation does not occur, levels of estrogen and progesterone remain low throughout the cycle and subjects should therefore be excluded from the analysis.

Seven of the studies reviewed did not employ a WSD, and only five studies performed hormone assays to confirm cycle phase and ovulation. Four of these five studies used a small sample size (no more than 11 subjects), and Marshall and colleagues (1983) compared two phases of presumed elevated sex steroids (days 8 to 10 versus days 22 to 24), rather than comparing a phase of low levels to one of high levels. The largest study (i.e., 24 subjects) to use a WSD and to confirm cycle phase and ovulation by blood assays found no statistically significant differences between the two tested cycle phases (i.e., days 3 to 5 versus days 19 to 23) (Mumenthaler et al. 1999).

In sum, critical review of the current literature suggests that the menstrual cycle is unlikely to affect alcohol pharmacokinetics and to account for gender differences, such as higher alcohol disappearance rates in women. Additional carefully designed research with appropriate controls may shed further light on this question.

### Performance Impairment

Few studies have investigated the effects of the menstrual cycle on alcohol-induced performance impairment (see table 4). Only one study has shown an interaction between the menstrual cycle phase and alcohol-related impairment. In that study, Jones and Jones (1978) concluded that gender-related differences in alcohol-induced performance impairment (alcohol dose: 0.52 g/kg) appear to be related to phases of the menstrual cycle for measures of reaction time. Four studies found psychomotor and cognitive performance tasks to be unaffected by the menstrual cycle while subjects were intoxicated (alcohol doses: 0.52 to 1.2 g/kg) (Brick et al. 1986; Jones 1975; Jones and Jones 1976*a*; Linnoila et al. 1980; Niaura et al. 1987).

## Influence of Sex Hormones

Testing women on different days of the menstrual cycle is only one way to investigate the relationship of sex hormones to drug action. Researchers have adopted other strategies, such as administering supplemental steroid hormones (animal studies), choosing ovariectomized or pregnant subjects, or recruiting females who use oral contraceptives (OCs). Some relevant findings from 14 studies are summarized below.

In animal studies, estrogen administration repeatedly increased hepatic ADH activity (Mezey et al. 1981; Teschke et al. 1986; Torres et al. 1985), and estrogen and progesterone administered together, but not estrogen alone, significantly increased BACs (Wanwimolruk and Levy 1987). Ovariectomy (surgical removal of the ovaries, leading to lower sex hormone levels) has not been accompanied by a change in BAC (Randall et al. 1981) or alcohol elimination rate (Mezey et al. 1981; Rachamin et al. 1980) in mice or rats. Teschke and colleagues (1986) found a significant decrease in hepatic ADH activity in ovariectomized rats; other researchers (Mezey et al. 1981) detected no such change in ADH function. Ovariectomy had no effect on gastric ADH of rats.

In some human studies, women taking OCs, which suppress the natural monthly hormonal cycle, reached significantly lower peak BACs and eliminated alcohol more slowly than women not taking OCs (Jones and Jones 1978; Zeiner and Kegg 1981; Jones and Jones 1984). Other researchers reported no effect of OC use on alcohol absorption, peak BAC, or elimination rates (Cole-Harding and Wilson 1987; Hay et al. 1984; Hobbes et al. 1985; Jeavons and Zeiner 1984; Niaura et al. 1987), although one investigation found significantly higher peak acetaldehyde concentrations in women using OCs (Jeavons and Zeiner 1984).

Two reviews concluded that women with elevated sex hormone levels, induced either by pregnancy or OCs, drink less alcohol (Zeiner and Kegg 1981).

The individual findings discussed above are consistent with the generalization that experimentally elevated estrogen levels appear to increase the activity of hepatic ADH, whereas ovariectomy (lower estrogen levels) tend to decrease hepatic ADH activity. Study results are ambiguous, however, and conclusions should be considered tentative.

## Conclusions

Women achieve higher BACs than do men after drinking equivalent amounts of alcohol, even when doses are adjusted for body weight. Women may be more susceptible than men to alcohol’s effects on cognitive functions (e.g., divided attention and memory). In contrast, impairment of psychomotor performance (e.g., eye-brain-hand coordination and body sway) does not seem to be affected by gender. Evidence suggests that men and women eliminate approximately the same total amount of alcohol per unit body weight per hour (i.e., same alcohol elimination rate), but that women eliminate significantly more alcohol per unit of lean body mass per hour (i.e., higher alcohol disappearance rate) than do men. Research has not determined whether this specific difference in disappearance rate may lead to faster recovery of alcohol-induced cognitive performance impairment in women. Additional studies might shed further light on this matter by testing subjects under heavy workload conditions (e.g., in-flight simulators) to increase the chance of identifying slight gender differences that may have escaped detection in previous studies.

Some data have prompted the speculation that hormonal fluctuations associated with the menstrual cycle might influence alcohol pharmacokinetics and alcohol’s effects on women. Critical review of the current literature, however, implies that the menstrual cycle is unlikely to affect alcohol pharmacokinetics and has little biological significance with respect to alcohol’s effects on performance.

## Figures and Tables

**Table 1:** Studies on Differences Between Men and Women in Alcohol Disappearance Rate

Study	*N*		Dose (g/kg)	β_60_

	Men	Women		
Taylor et al. 1996	11	12	0.77 M; 0.67 W	M < W*
Ammon et al. 1996	6	6	0.3	M < W*
Thomasson 1995	45	45	0.6 M; 0.5 W	M < W*
Thomasson et al. 1995	56	56	0.54^c^	M < W*
Smith et al. 1993	11	9	0.8	M < W*
Frezza et al. 1990	14	17	0.3	M ~ W
Mishra et al. 1989	9	9^a^	0.6	M < W*
Sutker et al. 1987*a*	10	8	0.50 or 0.76	M < W*
Cole-Harding and Wilson 1987	75	63	0.8^d^	M < W*
Holtzman et al. 1985	7	5	45.0 g/day, fixed	M < W*
Martin et al. 1985	194	208^b^	0.75	M ~ W
Arthur 1984	10	10	0.5	M ~ W
Jones and Jones 1976*a*	10	20	0.5	M ~ W

β_60_ = decrease of blood alcohol concentration (BAC) during concentration-independent phase of alcohol elimination (i.e., rate of disappearance); g/day = grams of alcohol per day; g/kg = grams of alcohol per kilogram of body weight; M = men; *N* = number of subjects; W = women; M < W* = significantly higher disappearance rate in women compared with men; M ~ W = no significant difference in disappearance rate.

a Study compared women with their male siblings.

b Total number of men and women reflect a mixture of monozygotic and dizygotic twin pairs, some same-sex pairs and some not.

c Average dose; individual doses were adjusted according to a formula to produce a BAC of 0.08 percent.

d Supplemental doses were administered to maintain peak BAC.

NOTE: Most of these studies show that women eliminate alcohol more quickly than men do, as measured by grams of alcohol per liter of blood per hour.

**Table 2:** Studies on Differences Between Men and Women in Post-Drink Performance

Study	*N*		Dose (g/kg)	BAC (%)	Tasks Tested	Difference	Remarks

	m	w					
Mulvihill et al. 1997	24	24	m 0.62	m 0.07	Reaction time	no	
			w 0.54	w 0.074			
Taylor et al. 1996	11	12	m 0.77	m 0.08	Flight simulator tasks	no	Performance change measured at acute intoxication and 8 hours later
			w 0.67	w 0.084			
Haut et al. 1989	22	22	0.80	Not reported	Word retrieval from long-term memory, cognitive decision tasks	yes	Greater performance deficits in women (responded significantly slower on all decision tasks)
Niaura et al. 1987	11	13	0.65	m 0.054	Divided attention, body sway, short-term memory, pursuit tracking	no	No overall gender differences in performance, but slower short-term memory recovery in women
				w 0.062		yes	
Mills and Bisgrove 1983	12	12	0.37	m 0.03	Divided attention, body sway	no	No gender differences at low alcohol dose, but women significantly more impaired on divided attention at high alcohol doses
			0.76^a^	w 0.03		yes	
				m 0.06			
				w 0.06			
Wait et al. 1982	20	20	1.25	Not reported	Eye-brain-hand coordination	no	
Burns and Moskowitz 1978	10	10	1.00	m 0.10	Information processing, hand steadiness, body sway, response latency	no	
				w 0.10			
Jones and Jones 1977	10	20	0.52	m 0.04	Immediate recall	yes	Women significantly more impaired on short-term memory
				w 0.04			
Jones and Jones 1976*a*,*b*	10	20	0.52	m 0.063	Immediate and delayed recall	yes	Women significantly more impaired on delayed recall, not on immediate recall.
				w 0.072			

BAC = blood alcohol concentration; g/kg = grams of alcohol per kilogram of body weight; *N* = number of subjects; m = men; w = women.

a with dose adjustments for different body fat percentage.

NOTE: These studies indicate that women show more impairment than men do on short-term cognitive tasks following alcohol consumption.

**Table 3:** Studies Showing a Significant Menstrual Cycle Effect on Alcohol Pharmacokinetics

Study	Absorption Rate	Peak BAC	Elimination Time	β_60_
Jones and Jones 1976*a*	21–28 > 13–18	21–28 > 1–3	—	
		21–28 > 13–18		
Kegg and Zeiner 1980		1 > 26		
Zeiner and Kegg 1981		1 > 24		1 > 24
King 1985	—	—	22 > 2	
Sutker et al. 1987*a*,*b*	—	—	20–25 > 2–7	20–25 > 2–7
			20–25 > 14	20–25 > 14

— = menstrual cycle had no effect on that specific pharmacokinetic variable; blank cells = no results were presented by the authors.

BAC = blood alcohol concentration; β_60_ = decrease of BAC during concentration-independent phase of alcohol elimination (i.e., rate of disappearance).

NOTES: Numbers in cells represent days or intervals of the menstrual cycle, adjusted to a 28-day cycle. Of 18 studies reviewed, only the 5 studies listed in this table found menstrual cycle effects on alcohol pharmacokinetics. Results of these five studies are inconsistent.

**Table 4:** Studies of Alcohol Effects on Performance in Relation to Menstrual Cycle

Study	*N*	Days of Cycle^a^	Dose (g/kg)	Task Tested	Cycle Effect^a^
Niaura et al. 1987	13	flw/mid/pre	0.65	Memory and attention	none
Brick et al. 1986	10	1–3/14–16/26–28	0.65	Memory, body sway, and divided attention	none
Linnoila et al. 1980	10	9/23	0.5/0.8/1.2	Reaction time and continuous tracking	none
Jones and Jones 1978	11	1–3/13–15/26–28	0.52	Reaction time	26–28 > 1–3 and 13–15
Jones 1975; Jones and Jones 1976*a*	14	1–3/13–18/21–28	0.52	Immediate and delayed memory	none

> = better performance; < = worse performance; flw = flow; g/kg = grams of alcohol per kilogram of body weight; mid = midcycle; *N* = number of subjects; pre = premenstrual.

a Days (or phases as indicated by the author) of the menstrual cycle at which testing took place, adjusted to a 28-day cycle.

b Numbers represent days or intervals of the menstrual cycle, adjusted to a 28-day cycle.

NOTE: Only one study demonstrated an interaction between menstrual cycle phase and alcohol-related impairment.

## Footnotes

1 The survey defined heavy alcohol use as the consumption of five or more drinks on the same occasion on each of 5 or more days in the past 30 days.

2 For definitions of moderate drinking, see Eckardt et al. 1998 and the article by Dufour on pp. 5–14; possible fetal effects of moderate drinking by pregnant women are discussed in the article by Jacobson and Jacobson, pp. 25–30.

3 Acetaldehyde is subsequently oxidized to acetate by an enzyme other than ADH. Acetate can be metabolized further and eliminated from the body or incorporated into various physiological substances.

4 A standard drink contains approximately 13.5 grams of alcohol (Eckardt et al. 1998) and is defined as 12 ounces of beer, 5 ounces of wine, or 1.5 ounces of distilled spirits.

5 A BAC of 0.10 percent (or less, depending on the State) is the legal limit for operating a motor vehicle.

6 A third, but quantitatively insignificant, pathway of alcohol metabolism involves the enzyme catalase, which converts alcohol to acetaldehyde and water.

7 Pursuit-tracking tasks provide a measure of eye-hand coordination related to steering a motor vehicle. From the public health standpoint, the key practical consideration emerging from the aforementioned research may perhaps be that women who choose to drink moderately should not rely on non-gender-specific guidelines for minimizing alcohol’s effects on mental functions.

8 Federal Aviation Administration regulations state that it is forbidden to pilot an aircraft while having a BAC of 0.04 percent or more.
